# Modular driveline cable exchange due to internal cable damage in a patient with HeartMate 3: a case report

**DOI:** 10.1093/ehjcr/ytag076

**Published:** 2026-01-30

**Authors:** So Sasaki, Sakae Takenaka, Toshiyuki Nagai, Toshihisa Anzai

**Affiliations:** Department of Cardiovascular Medicine, Faculty of Medicine and Graduate School of Medicine, Hokkaido University, Kita 15, Nishi 7, Kita-ku, Sapporo, Hokkaido 060-8638, Japan; Department of Cardiovascular Medicine, Faculty of Medicine and Graduate School of Medicine, Hokkaido University, Kita 15, Nishi 7, Kita-ku, Sapporo, Hokkaido 060-8638, Japan; Department of Cardiovascular Medicine, Faculty of Medicine and Graduate School of Medicine, Hokkaido University, Kita 15, Nishi 7, Kita-ku, Sapporo, Hokkaido 060-8638, Japan; Department of Cardiovascular Medicine, Faculty of Medicine and Graduate School of Medicine, Hokkaido University, Kita 15, Nishi 7, Kita-ku, Sapporo, Hokkaido 060-8638, Japan

## Case description

**Figure 1:**
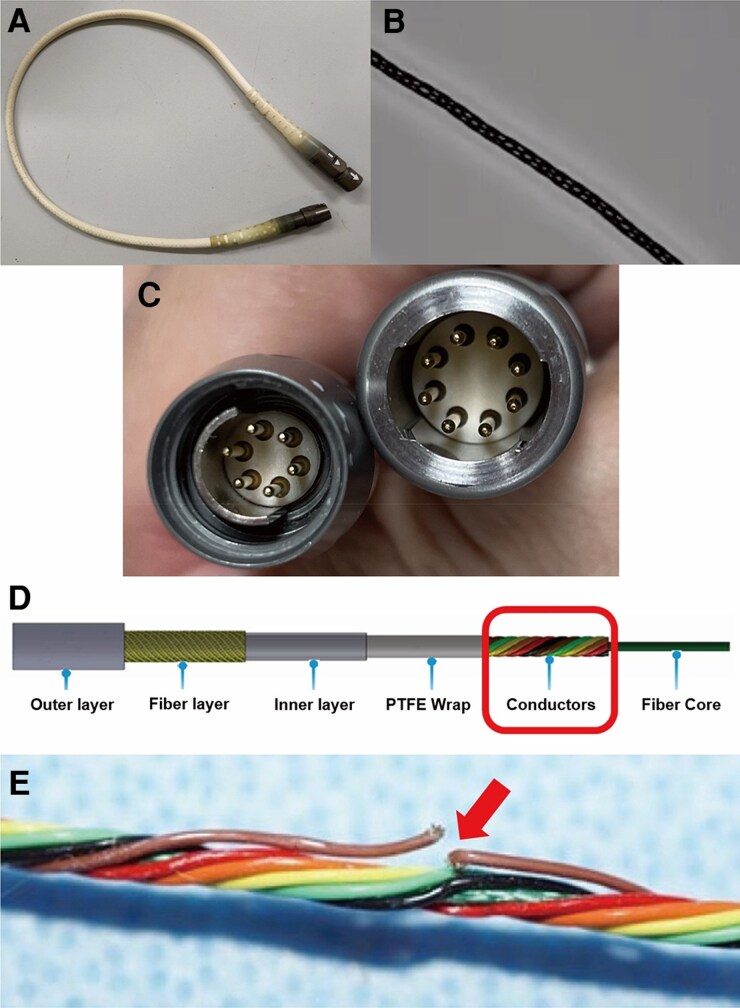
Modular driveline cable in a patient with HeartMate 3. (*A*) Outer layer of the modular cable. (*B*) Modular cable under radiographic imaging. (*C*) Connector between the modular and pump cables. (*D*) Driveline structure (images provided by Abbott Laboratories). PTFE, polytetrafluoroethylene. (*E*, arrow) Internal conductor damage in the modular cable (images provided by Abbott Laboratories).

A 62-year-old woman with non-ischaemic dilated cardiomyopathy underwent implantation of a HeartMate 3 (HM3) left ventricular assist device (LVAD) (Abbott Laboratories, North Chicago, IL, USA) as a bridge to heart transplantation. After 4.5 years with LVAD support, she was admitted to our hospital because of a ‘Driveline Power Fault’ alarm. The LVAD demonstrated adequate function, with a speed of 4600 rpm, flow rate of 3.6 L/min, power of 3.0 W, and pulsatility index of 7.5. Haemodynamic status was stable (blood pressure 82/66 mmHg, heart rate 63 b.p.m.). The alarm occurred intermittently regardless of the body position during hospitalization. Initial evaluation by the manufacturer suggested a driveline connection failure between the cables or severe damage to the driveline or system controller. First, we examined the driveline cable by direct visual examination (*Figure 1A*), radiographic imaging (*Figure 1B*), and inspection of the connector between the modular and pump cables (*Figure 1C*); however, no abnormalities were observed. Second, the system controller was replaced. Nevertheless, the alarm recurred. Given these findings, we strongly suspected damage to the driveline cables and performed a modular cable exchange. No further alarms were reported following cable exchange, and the patient was discharged without LVAD pump exchange. Examination of the disassembled modular cable revealed a complete disruption of an internal conductor segment (*Figures 1D* and *E*).

The HM3 driveline, which includes duplicate sets of three conductors and a more flexible outer layer, represents an improvement over that of the HeartMate II. Although some cases requiring modular cable exchange have been reported,^1,2^ internal conductor damage within the modular cable has not previously been described in patients with HM3. In the present case, no external factors were identified; however, the same segment of modular cable was repeatedly bent during the prolonged waiting period for heart transplantation, which might have caused complete disruption. To the best of our knowledge, this is the first reported case of modular cable exchange due to internal conductor damage in a patient with HM3. When a ‘Driveline Power Fault’ alarm occurs, internal cable damage should be considered even in the absence of external abnormalities.

## Data Availability

No new data were generated or analysed in support of this research.
